# The Surgical Treatment of Spastic Infantile Paralysis

**Published:** 1902-09-13

**Authors:** 


### THE SURGICAL TREATMENT OF SPASTIC INFANTILE PARALYSIS.

When infantile spastic paralysis has reached the stage of contracture it is to be feared that there is not much to be done by merely medical means. Massage and electricity may do something to preserve warmth and encourage circulation in the limbs, and perhaps to prevent further advance of the muscular contraction, but that is all. Help even from surgery has been despaired of by many, and so long as surgery devoted itself mainly to the division of contracted tendons the results certainly were not encouraging. The matter, however, is somewhat different now that tendon transplantation is called to one's aid, and operation is followed by a prolonged treatment by specially devised splints. Mr. Robert Jones, of Liverpool, speaking at Manchester, said that he had now operated upon over a hundred cases of spastic paralysis, and that notwithstanding the pessimism of the text books, he would argue from his experience that a large proportion of children suffering from severe spastic paralysis may be improved both in body and mind by surgical methods, enabled to walk with comparatively little deformity, many requiring only the aid to be derived from one or two sticks, and thus transformed into useful members of the community. A class which we must place outside remedial art includes the idiot, the microcephalic, and the violent irritable type of diplegic so often seen subject to fits and active athetotic movements, who has generally lost all control over his excretions; but the treatment of any condition short of this may be undertaken with varying success. Another class of case, however, which gives rise to great anxiety and trouble is that in which the affection of the hands is of such a kind as to give but slight hope of their being able to afford much assistance to the lower limbs during walking.

The surgical treatment which Mr. Robert Jones advocates consists of a combination of tendon division, tendon transplantation (transplantation of contracted flexors into extensors) and the taking of infinite pains in splinting and keeping the limbs for several months after the operation in positions opposed to those occupied by them in their contracted state. Into the details we cannot now enter, but must content ourselves with indicating the extent of the improvement which, according to Mr. Jones, we may hope for in this distressing class of cases. "I have operated," he says, "on cases from 12 months to 20 years of age. A large number of these were so bad that they had never attempted to place one foot before the other. Some were structurally flexed (contractured) at the ankle, knee, and hip. A most helpless youth of 20, one limb across the other, was able in six months to stand erect, and walk with two sticks, and 12 months later could move his limbs north, south, east, and west with hardly an appreciable jerk." The mental condition of the children obviously improves when their physical defects are remedied, and they are enabled to mix with their little friends. Complete recovery in spastic paraplegia is of course impossible.

